# Intracellular Serine Protease Inhibitor SERPINB4 Inhibits Granzyme M-Induced Cell Death

**DOI:** 10.1371/journal.pone.0022645

**Published:** 2011-08-03

**Authors:** Pieter J. A. de Koning, J. Alain Kummer, Stefanie A. H. de Poot, Razi Quadir, Roel Broekhuizen, Anne F. McGettrick, Wayne J. Higgins, Bart Devreese, D. Margaret Worrall, Niels Bovenschen

**Affiliations:** 1 Department of Pathology, University Medical Center Utrecht, Utrecht, The Netherlands; 2 School of Biomolecular and Biochemical Science, University College Dublin, Dublin, Ireland; 3 Laboratory for Protein Biochemistry and Biomolecular Engineering, Department of Biochemistry and Microbiology, Ghent University, Ghent, Belgium; Monash University, Australia

## Abstract

Granzyme-mediated cell death is the major pathway for cytotoxic lymphocytes to kill virus-infected and tumor cells. In humans, five different granzymes (*i.e.* GrA, GrB, GrH, GrK, and GrM) are known that all induce cell death. Expression of intracellular serine protease inhibitors (serpins) is one of the mechanisms by which tumor cells evade cytotoxic lymphocyte-mediated killing. Intracellular expression of SERPINB9 by tumor cells renders them resistant to GrB-induced apoptosis. In contrast to GrB, however, no physiological intracellular inhibitors are known for the other four human granzymes. In the present study, we show that SERPINB4 formed a typical serpin-protease SDS-stable complex with both recombinant and native human GrM. Mutation of the P2-P1-P1′ triplet in the SERPINB4 reactive center loop completely abolished complex formation with GrM and N-terminal sequencing revealed that GrM cleaves SERPINB4 after P1-Leu. SERPINB4 inhibited GrM activity with a stoichiometry of inhibition of 1.6 and an apparent second order rate constant of 1.3×10^4^ M^−1^s^−1^. SERPINB4 abolished cleavage of the macromolecular GrM substrates α-tubulin and nucleophosmin. Overexpression of SERPINB4 in tumor cells inhibited recombinant GrM-induced as well as NK cell-mediated cell death and this inhibition depended on the reactive center loop of the serpin. As SERPINB4 is highly expressed by squamous cell carcinomas, our results may represent a novel mechanism by which these tumor cells evade cytotoxic lymphocyte-induced GrM-mediated cell death.

## Introduction

Cytotoxic T lymphocytes (CTLs) and natural killer (NK) cells (*i.e.* cytotoxic lymphocytes) play a pivotal role in the effector arm of the immune response that eliminate virus-infected cells and tumor cells [1]. Cytotoxic lymphocytes predominantly destroy their target cells by releasing the content of their cytolytic granules. These granules contain perforin and a family of unique structurally homologous serine proteases known as granzymes [2]. While perforin facilitates the entry of granzymes into the target cell, the latter induce cell death by cleaving critical intracellular substrates [3].

In humans, five different granzymes (GrA, GrB, GrH, GrK, and GrM) are known that differ on the basis of their substrate specificity [4]. All granzymes induce cell death with partially overlapping morphological hallmarks [4]. While GrA and GrB have been extensively studied, far less is known about the molecular cell death mechanisms of the other human granzymes [5]. Recently, it has been demonstrated that GrM, which is highly expressed by NK cells, NKT cells, γδ-T cells, and CD8^+^ effector T cells [6], [7], [8], mediates a major and novel perforin-dependent cell death pathway that plays a significant role in cytotoxic lymphocyte induced death [9]. In tumor cell lines, GrM directly and efficiently cleaves a diverse set of substrates, *i.e.* ICAD, PARP, HSP75, ezrin, α-tubulin, PAK 2, survivin, and nucleophosmin [10], [11], [12], [13], [14].

Tumor cells can escape from cytotoxic lymphocyte-induced killing by expression of cell death inhibitors in their cytoplasm, like the caspase-inhibitors XIAP and FLIP [15], [16], and the GrB-inhibitor SERPINB9 (PI9) [17]. SERPINB9 is the only known intracellular human granzyme inhibitor and protects against GrB-induced apoptosis [17], [18]. Expression of SERPINB9 is associated with a poor clinical outcome in various types of tumors (*e.g.* lymphomas and melanomas) [19], [20], [21]. SERPINB9 belongs to the intracellular (B-clade) sub-family of human serine protease inhibitors (serpins). Serpins share a unique inhibitory mechanism. Upon cleavage by a specific target protease in their reactive center loop (RCL), serpins undergo a conformational change after which the serpin and the target protease are covalently bound, leaving the latter kinetically inactive [22].

In contrast to GrB, no physiological intracellular inhibitors are known for the other four human granzymes [23]. Since GrM is a very potent specialized inducer of tumor cell death [5], [24] and plays an important role in anti-tumor function *in vivo* [25], we aimed to identify an intracellular inhibitor of human GrM. In the present study, we demonstrate that SERPINB4 [also called squamous cell carcinoma antigen 2 (SCCA-2) or leupin] directly inhibits human GrM proteolytic activity and that overexpression of SERPINB4 in HeLa cells inhibits recombinant GrM-induced as well as NK cell-mediated cell death. This may represents a novel mechanism by which tumor cells evade GrM-mediated killing by cytotoxic lymphocytes.

## Materials and Methods

### Recombinant proteins

Expression and purification of recombinant human GrM and the catalytically inactive GrM-SA variant was performed as described previously [10]. Briefly, cDNA encoding mature human GrM (residues Ile^26^-Ala^257^) was cloned into the yeast expression vector pPIC9 (Invitrogen, Paisley, UK). Catalytically inactive GrM-SA, in which the Ser^195^ residue in the catalytic center is replaced by Ala, was generated by site-directed mutagenesis (Stratagene, Cedar Creek, TX). Plasmids were transformed into the GS115 strain of P. pastoris (Invitrogen) and granzymes were expressed in conditioned media for 72 h. Recombinant GrM and GrM-SA were purified to homogeneity by cation-exchange chromatography (GE Healthcare, Diegem, Belgium) and dialyzed against 50 mM Tris (pH 7.4) and 150 mM NaCl. Recombinant GrM, but not GrM-SA, was active as determined by a synthetic chromogenic leucine substrate (Bachem, Weil am Rhein, Germany).

Expression and purification of recombinant SERPINB4 wild type and SERPINB4 RCL-mutant was performed using the expression vector pRSETC (Invitrogen) as described previously [26]. SERPINB4, coding an N-terminal His_6_-tagged fusion protein, and SERPINB4 RCL-mutant, in which the P2-Glu^353^, P1-Leu^354^, P1′-Ser^355^ amino acids were mutated into P2-Gln^353^, P1-Gly^354^, P1′-Ala^355^, were expressed in E.coli BL21 (DE3) using Overnight Express auto inducing medium (Merck, Nottingham, UK) containing 100 µg/ml ampicillin. Following growth at 37°C for 24 h, cells were harvested by centrifugation at 15,000 g for 30 minutes and lysed using Bugbuster (Merck) lysis reagent. Soluble material was clarified by centrifugation of the lysate at 21,000 g for 30 minutes at 4°C. The recombinant serpin was purified using a His-Bind Purification kit (Merck). Pooled imidazole eluted fractions were buffer exchanged into 50 mM Tris pH 8.0 and recombinant protein was stored at −80°C until required. Concentrations of the purified proteins were measured according to the procedure of Bradford (Bio-Rad, Hercules, CA).

### Generation of a novel mAb against human GrM

**Figure 1:**
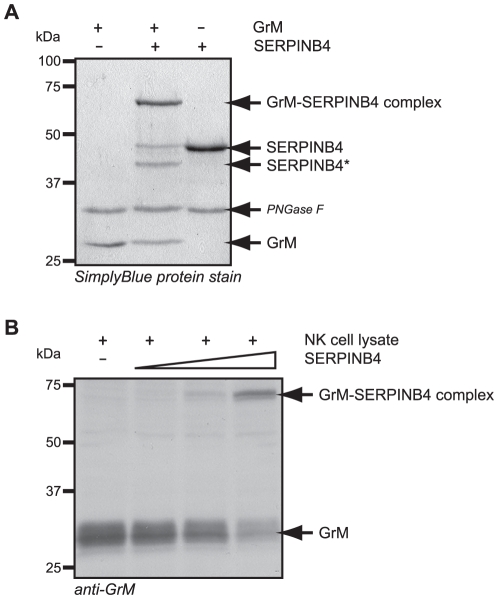
SERPINB4 forms a typical serpin-protease SDS-stable complex with recombinant and native human GrM. (**A**) Purified recombinant GrM (1.8 µM) and SERPINB4 (1.8 µM) were incubated for 1 h at 37°C. All samples were treated with PNGase F, separated by SDS-PAGE, and analyzed by SimplyBlue staining. (**B**) Cell lysate of the NK cell line KHYG-1 (33 µg) was incubated with recombinant SERPINB4 (0, 50, 150, and 450 ng) for 2 h at 37°C. Subsequently, samples were immunoblotted for native GrM. GrM, SERPINB4, GrM-SERPINB4 complexes, and cleaved SERPINB4 (SERPINB4*) are indicated.

Mice were immunized with purified recombinant human GrM. Obtained hybridomas were screened for antibodies that reacted with GrM in different applications as previously described [27]. Anti-GrM mAb 3D4D7 (IgG1 isotype) was highly sensitive against Western-blotted recombinant GrM as well as native GrM from NK cell lysates (Fig. 1). Cross-reactivity of mAb 3D4D7 against recombinant human GrB and GrK was excluded (data not shown).

### Cell culture and transfection

293T and Jurkat cells were maintained in DMEM and RPMI-1640 medium (Invitrogen), respectively, supplemented with 10% (v/v) heat-inactivated fetal bovine serum (Sigma), 100 U/ml penicillin, and 100 µg/ml streptomycin (Invitrogen). The human NK cell line KHYG-1 [28] was purchased from the Health Science Research Resources Bank (JCRB0156) of the Japan Health Sciences Foundation and cultured similarly to Jurkat cells, with the addition of 50 ng/ml recombinant human interleukin 2 (Wako, Osaka, Japan). The previously described HeLa cell lines, stably transfected with pcDNA3 SERPINB4, pcDNA3 SERPINB4 RCL-mutant, or pcDNA3 vector only [26], were cultured similarly to 293T cells, with the addition of G418 (0.5 mg/ml). The plasmid pEGFP-N1 containing C-terminal green fluorescent protein (GFP)-tagged SERPINB4 (kindly provided by Dr. Wun-Shaing W. Chang, National Institute of Cancer Research, National Health Research Institutes, Taiwan, ROC) or mock control vector were transiently transfected into 293T cells using linear polyethylenimine (PEI) (Polysciences, Warrington, PA), according to the manufacturer's instructions. Cell lysates were prepared by three freezing-thawing cycles in liquid nitrogen of cells resuspended in a buffer containing 20 mM Tris (pH 7.4) and 150 mM NaCl. Protein concentrations of the supernatants were measured according to the procedure of Bradford.

### Analysis of complex formation by SDS-PAGE and immunoblotting

Indicated amounts of purified recombinant SERPINB4 and GrM proteins were incubated in 20 mM Tris (pH 7.4) and 150 mM NaCl for 1 h at 37°C. When noted, Jurkat or KHYG-1 cell lysate was incubated with the recombinant protein(s) for the indicated times at 37°C. Samples containing recombinant proteins only were additionally treated with PNGase F (New England BioLabs, Ipswich, MA), according to the manufacturer's instructions. Subsequently, samples were separated on 10% SDS-PAGE gels under reducing conditions. For SDS-PAGE analysis, gels were stained with SimplyBlue (Invitrogen), according to the manufacturer's instructions. For immunoblot analysis, proteins were transferred onto immobilon–P membranes (Millipore, Billerica, MA). After blocking with 5% (w/v) Marvel dried skimmed milk (Premier International Foods, Coolock, UK) in TBS-T (10 mM Tris-HCl, pH 8.0, 150 mM NaCl, 0.1% Tween-20), membranes were incubated for two hours at RT with our novel mouse anti-human GrM mAb (clone 3D4D7; 1.0 µg/ml), mouse anti-human α-tubulin mAb (clone B-5-1-2; 0.4 µg/ml) (Sigma), mouse anti-human nucleophosmin (clone FC-61991; 2.0 µg/ml) (Invitrogen), rabbit anti-human nm23H-1 (clone C-20:sc-343; 1.0 µg/ml) (Santa Cruz Biotechnology, Santa Cruz, CA), or mouse anti-human β-actin mAb (clone 2A2.1) (US Biological, Swampscott, MA). Goat anti-mouse IgG+IgM HRP (Biosource, Camarillo, CA) conjugate was used as secondary antibody. Bound antibodies were visualized using 3,3′-diaminobenzidine (DAB) (0.6 mg/ml) or enhanced chemiluminescence substrate (ECL) (GE Healthcare).

### Immunoprecipitation

Cell lysates of mock and GFP-tagged SERPINB4 transfected 293T cells were incubated with 10 µg/ml anti-GFP mAb (Roche, Mannheim, Germany) and protein A/G plus-agarose (Santa Cruz Biotechnology), and rotated end-over-end in non-sticky microfuge tubes (Ambion, Austin, TX) for 16 h at 4°C. Precipitates were washed three times with 50 mM Tris (pH 7.4), 150 mM NaCl and incubated with 2.3 µM rh-GrM for 1 h at 37°C. Next, the samples were washed twice more and finally boiled in reducing Laemmli sample buffer. Immunoblotted samples were stained by Coomassie Brilliant Blue (BioRad) and the cleavage-product of interest was excised and identified by Edman degradation, using a 476A protein sequencer (Applied Biosystems, Foster City, CA).

### Stoichiometry of inhibition

Increasing concentrations (0–3 µM) of recombinant SERPINB4 was incubated with a constant amount of recombinant human GrM (2 µM) in 50 mM Tris (pH 7.4) and 150 mM NaCl for 2 h at 37°C. Samples were diluted 10-fold in 100 mM Tris (pH 7.4), 200 mM NaCl, and 0.01% Tween containing 1 mM synthetic chromogenic leucine substrate (AAPL-pNA) (Bachem) to terminate the inhibition reactions and transferred to a microplate (Greiner, Kremsmunster, Austria) to assay residual GrM activity. The velocity of substrate hydrolysis by residual active GrM was measured at A_405_ using a microtiter plate reader (Anthos, Cambridge, UK). The fractional activity (velocity of GrM with a serpin/velocity of GrM only) was plotted against the ratio of [serpin]_0_/[GrM]_0_. Linear regression analysis was used to determine the x-axis intercept as a value for the stoichiometry of inhibition.

### Apparent second order rate constant

The interaction of SERPINB4 with GrM was determined under second order conditions [29]. Recombinant GrM was incubated with recombinant SERPINB4 at equimolar concentrations. Samples of this reaction were taken after 30, 60, 120, 180, 240 and 360 seconds and directly diluted 20-fold in 100 mM Tris (pH 7.4), 200 mM NaCl, and 0.01% Tween containing 1 mM of the chromogenic substrate AAPL-pNA. The rate of substrate hydrolysis by GrM was measured in time at A_405_ using a microtiter plate reader (Anthos). Since serpin-serine protease interactions are irreversible, velocities of the substrate hydrolysis were converted to residual active GrM concentrations using a GrM concentration standard curve incubated without SERPINB4. The rate of change in the amount of residual active GrM over time is described in equation (1), where the slope of the plot of reciprocal residual active GrM over time yields the apparent second order rate constant (*k_inh_*).
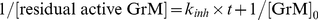
(1)


### RT-PCR and immunoblot analysis for SERPINB4 expression

RNA was isolated from (stably transfected) HeLa cells using RNA isolator™ (Genosys). RNA (1–5 µg) was reverse transcribed using MMLV-reverse transcriptase, and the resulting cDNA was used for RT-PCR amplification. The following primers were used: GAPDH-sense 5′-TGAAGGTCGGAGTCAACG-3′, GAPDH-antisense 5′-CATGTGGGCCATGAGGTC-3′, SERPINB4-sense 5′-GGGGGATCCATATGAATTCACTCAGTGAAG-3′, and SERPINB4-antisense 5′-CCCCCGGGTACCTACGGGGATGAGAATCTG-3′. Immunoblot analysis for SERPINB4 protein expression by (stably transfected) HeLa cells was performed as previously described [26].

### Granzyme M killing assays

HeLa cells stably transfected with SERPINB4 or SERPINB4 RCL-mutant were grown to confluence in a 96-well tissue-culture plate. Cells were washed twice in serum-free DMEM, after which they were incubated at 37°C with a sublytic dose of streptolysin O (SLO) (0.5 µg/ml) (Sigma) and indicated concentrations of granzyme in serum-free DMEM for 30 min. Cells were washed twice with supplemented DMEM, after which cells were incubated for indicated periods of time at 37°C. For the methylene blue assay, cells were carefully washed with PBS and remaining adherent cells were fixed with methanol for 30 min at room temperature. After drying, cells were stained with 0.05% (w/v) methylene blue dye (Merck) in borate-buffered saline (pH 8.4) for 15 min, washed 3 times with water, and air dried. Bound dye was dissolved in 0.1 N HCl (100 µl/well) and measured photospectrometrically at 630 nm. To assess cell viability by flow cytometry, cells were stained with AnnexinV-fluos (Invitrogen) and propidium iodide (PI) for 15 min in a buffer containing 140 mM NaCl, 4 mM KCl, 0.75 mM MgCl_2_, 1.5 mM CaCl_2_, and 10 mM HEPES (pH 7.4). Flow cytometry was performed on a FACSCalibur (BD Biosciences) and data was analyzed using CellQuest Pro software (BD Biosciences).

### NK-cell mediated cytotoxicity assay

HeLa cells stably transfected with SERPINB4 or SERPINB4 RCL-mutant were grown to confluence in a 96-well tissue-culture plate and loaded for 10 min with 10 µM of the fluorescent cell staining dye carboxyfluorescein diacetate succinimidyl ester (CFDA-SE). These HeLa cells were co-cultured with KHYG-1 NK cells in varying E∶T ratio's for 16 h at 37°C. Cells were stained with PI and analyzed by flow cytometry. During data analysis, a gate on CFDA-SE positive cells was used to distinguish between KHYG-1 and HeLa cells. The percentage of living untreated HeLa cells was set at 100% and the percentages of treated cells were matched correspondingly, after which the percentage of specific cytotoxicity was calculated.

### Statistical analysis

All statistical analyses were performed using the Student's t-test.

## Results

### SERPINB4 forms a typical serpin-protease SDS-stable complex with human GrM

Upon cleavage by a specific target protease, most serpins form SDS-stable covalent-bound complexes with that protease, in which the latter one becomes inactive [22]. Since human GrM displays a restricted cleavage site specificity in that it predominantly cleaves after P1-Leu [10], [11], [12], [30] and that SERPINB4 is the only human intracellular B-clade serpin that harbours a Leu at the putative P1-position in its RCL [31], [32], we investigated complex formation of SERPINB4 with GrM by SDS-PAGE analysis using purified recombinant proteins (Fig. 1A). Purified recombinant GrM and SERPINB4 migrated with a molecular mass of 28 and 44 kDa, respectively (Fig. 1A, left and right lane). Incubation of GrM with SERPINB4 revealed an SDS-stable complex band with the expected molecular mass of around 70 kDa (Fig. 1A, middle lane). The majority of SERPINB4 molecules formed a complex with GrM, whereas a small fraction of SERPINB4 was cleaved by GrM without trapping this protease. Next, we studied whether SERPINB4 could also bind to and form an SDS-stable complex with native human GrM (Fig. 1B). The human NK cell line KHYG-1 was used as a source for native GrM. Incubation of a KHYG-1 cell lysate with recombinant SERPINB4 and subsequent immunoblotting for the protease revealed the formation of a complex between native GrM and recombinant SERPINB4 in a concentration-dependent manner (Fig. 1B). These data demonstrate that SERPINB4 forms a classical SDS-stable serpin-protease complex with both recombinant and native human GrM.

### GrM cleaves SERPINB4 in its RCL after P1-Leu

**Figure 2:**
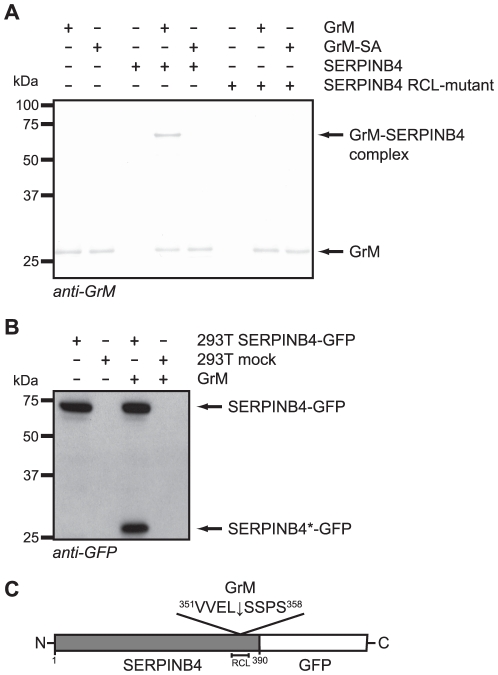
Mutation of the SERPINB4 RCL at the P2-P1-P1′ positions completely abolishes complex formation with human GrM. (**A**) A RCL-mutant of SERPINB4 was employed in which the amino acids at the putative P2(Glu^353^)-P1(Leu^354^)-P1′(Ser^355^) positions were mutated into P2(Gln^353^)-P1(Gly^354^)-P1′(Ala^355^). Purified recombinant GrM (0.9 µM), GrM-SA (0.9 µM), SERPINB4 (0.9 µM), and SERPINB4 RCL-mutant (0.9 µM) were incubated for 1 h at 37°C. All samples were treated with PNGase F, separated by SDS-PAGE, and immunoblotted for GrM. Bound antibodies were visualized using DAB. (**B**) Cell lysates of 293T cells transfected with C-terminal GFP-conjugated SERPINB4 or an empty vector (mock) were incubated with recombinant GrM (0.5 µM) for 1 h at 37°C. Subsequently, samples were immunoblotted for GFP. SERPINB4-GFP represents the full length protein, whereas SERPINB4*-GFP depicts the C-terminal cleavage product. (**C**) Schematic representation of C-terminal GFP-conjugated SERPINB4, including the GrM cleavage site after Leu^354^ at the P1-position in the RCL.

To investigate if complex formation of SERPINB4 with GrM depends on the RCL and corresponding conformational change of the serpin, a RCL-mutant of SERPINB4 was employed in which the amino acids at the putative P2(Glu^353^)-P1(Leu^354^)-P1′(Ser^355^) positions were mutated into P2(Gln^353^)-P1(Gly^354^)-P1′(Ala^355^). Western blot analysis for GrM revealed a complex with a molecular mass of about 70 kDa when the purified recombinant wild type proteins of GrM and SERPINB4 were incubated (Fig. 2A). In contrast, no complex formation was detected upon incubation of SERPINB4 RCL-mutant with GrM. Furthermore, a mutated catalytically inactive counterpart of GrM was included (GrM-SA). As expected, this GrM-SA was not able to form a complex with SERPINB4 (Fig. 2A). Thus, complex formation of SERPINB4 with GrM appears to depend on the RCL of the serpin and the catalytic triad of the protease.

Next, the cleavage site of GrM in the RCL of SERPINB4 was determined. Therefore, cell lysates of 293T cells expressing either C-terminal GFP-tagged full length SERPINB4 or an empty vector were incubated with GrM. Immunoblotting for GFP showed a C-terminal cleavage product of SERPINB4 (SERPINB4*-GFP) with the expected molecular mass of about 29 kDa (Fig. 2B), as the RCL of SERPINB4 is near its C-terminus and the molecular mass of the linked GFP is about 25 kDa. Immunoprecipitation of GFP-conjugated SERPINB4 from 293T cell lysate and N-terminal sequencing of the subsequent GrM-induced C-terminal cleavage fragment revealed that GrM indeed cleaves SERPINB4 in its RCL, at least after the amino acid Leu^354^ at the P1-position in the sequence ^351^VVEL↓SSPS^358^ (Fig. 2C).

### Stoichiometry of inhibition of GrM and SERPINB4

**Figure 3:**
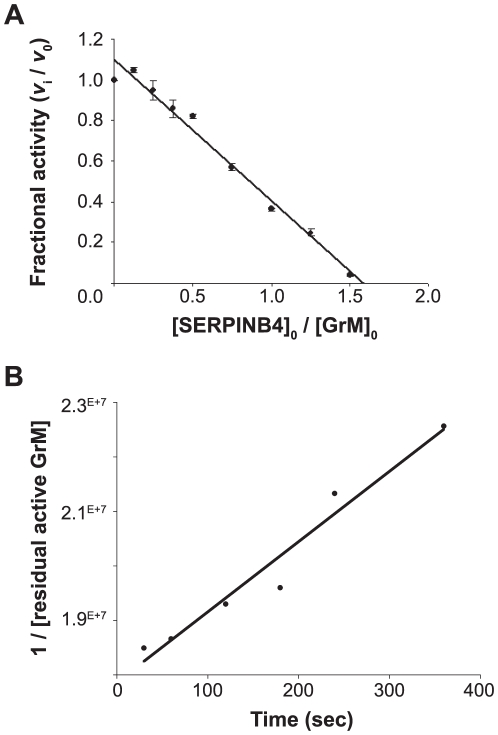
Kinetic analyses of GrM-inhibition by SERPINB4. (**A**) Purified recombinant human GrM (2 µM) was incubated with different concentrations of recombinant SERPINB4 (0–3 µM) for 2 h at 37°C. Residual GrM activity was monitored by addition of a synthetic chromogenic leucine substrate (1 mM) and measuring A_405_ in time. The fractional activity (velocity of substrate hydrolysis by GrM in the presence of SERPINB4/velocity of substrate hydrolysis by GrM without SERPINB4) was plotted against the ratio of [SERPINB4]_0_/[GrM]_0_. Linear regression analysis was used to calculate the x-intercept as a value for the SI and determined to be 1.6±0.07 (mean ± SD of 4 independent experiments). (**B**) The apparent second order rate constant (*k_inh_*) of the inhibition of GrM by SERPINB4 was determined under second order conditions. Equimolar concentrations of recombinant human GrM and SERPINB4 were incubated. Aliquots were removed at different time points and the reaction was directly stopped by 20-fold dilution with buffer containing a synthetic chromogenic leucine substrate (1 mM). Residual GrM activity was determined by measuring the velocity of substrate hydrolysis in time at A_405_. These velocities were converted to residual active GrM concentrations using a GrM concentration standard curve. The apparent second order rate constant (*k_inh_*) was calculated from the slope of the plot of reciprocal residual active GrM over time and determined to be 1.3×10^4^ M^−1^s^−1^ (graph indicates one representative example of three independent experiments with similar results).

The interaction of a serpin with its physiological target protease usually results in complex formation with a stoichiometry of inhibition (SI) value close to 1. The SI value indicates how many serpin-molecules are needed to inhibited one molecule of target protease. If a serpin is also a substrate in parallel to an inhibitor of its target protease, this is reflected by a SI value greater than 1. The SI value for inhibition of GrM by SERPINB4 was determined by measurement of residual GrM activity at increasing [SERPINB4]_0_/[GrM]_0_ ratios (Fig. 3A). Complete inhibition of GrM activity was obtained at a [SERPINB4]_0_/[GrM]_0_ ratio of 1.6±0.07 (mean ± SD of 4 independent experiments) (Fig. 3A). This indicates that about three molecules of SERPINB4 are needed to inhibit two GrM molecules.

### Kinetic analysis of the inhibition of GrM activity by SERPINB4

To determine the rate of complex formation between GrM and SERPINB4, the apparent second order rate constant (*k_inh_*) was measured under second order conditions. Upon incubation of recombinant GrM with equimolar concentrations of SERPINB4 samples were taken in time to measure the residual GrM-activity. Addition of a synthetic chromogenic leucine substrate to these aliquots revealed that the concentration of active GrM decreased linear in time (Fig. 3B). The apparent second order rate constant (*k_inh_*) was calculated from the slope of the plot of reciprocal residual active GrM over time and was determined to be 1.3 (±0.05)×10^4^ M^−1^s^−1^ (mean ± SD of 3 independent experiments). This *k_inh_* was not affected by inclusion of the glycosaminoglycan heparin in these experiments, which is a known co-factor of several serpins that enhances the *k_inh_* towards their target proteases [33] (data not shown).

### SERPINB4 inhibits cleavage of natural macromolecular substrates by GrM

**Figure 4:**
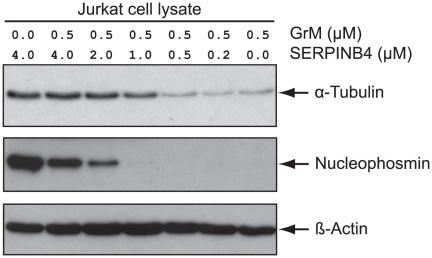
SERPINB4 inhibits GrM-mediated cleavage of macromolecular substrates. Indicated concentrations of recombinant GrM and SERPINB4 were incubated for 2 h at 37°C. Jurkat tumor cell lysate (2 µg) was added to the samples and incubated for another 4 h at 37°C to determine the residual GrM-activity towards macromolecular substrates. Finally, all samples were immunoblotted for α-tubulin, nucleophosmin, and β-actin.

The inhibitory effect of SERPINB4 on the activity of human GrM was further investigated using natural macromolecular substrates of GrM. GrM and SERPINB4 were incubated at various concentration-ratios and GrM-activity towards two known direct GrM-substrates [10], [11] in a Jurkat tumor cell lysate was determined by immunoblotting (Fig. 4). As expected, in the absence of SERPINB4, human recombinant GrM efficiently cleaved the 55 kDa α-tubulin subunit as well as the 37 kDa nucleolar phosphoprotein nucleophosmin. Recombinant SERPINB4 impaired cleavage of both α-tubulin and nucleophosmin by GrM in a concentration-dependent manner (Fig. 4). Excessive concentrations of SERPINB4 over GrM completely inhibited GrM activity towards α-tubulin, whereas some nucleophosmin cleavage was still observed, suggesting that nucleophosmin is a more efficient GrM-substrate than α-tubulin. β-actin served as a loading control (Fig. 4). In conclusion, SERPINB4 inhibits GrM-mediated cleavage of the natural macromolecular substrates α-tubulin and nucleophosmin in a tumor cell lysate.

### Overexpression of SERPINB4 inhibits recombinant GrM-induced as well as NK cell-mediated cell death

**Figure 5:**
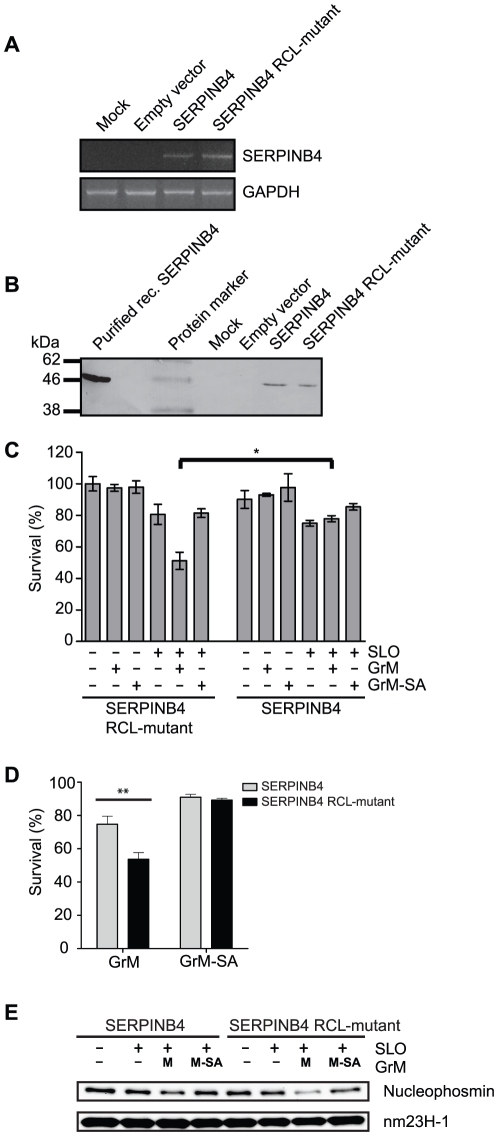
Overexpression of SERPINB4 in HeLa cells inhibits GrM-induced cell death. (**A**) RT-PCR analysis of stably transfected HeLa cells for SERPINB4 and GAPDH mRNA expression (upper and lower panel, respectively). (**B**) Immunoblot analysis of SERPINB4 protein expression by HeLa cells stably transfected with pcDNA3 SERPINB4, pcDNA3 SERPINB4 RCL-mutant or pcDNA3 empty vector. (**C**) HeLa cells stably transfected with SERPINB4 or SERPINB4 RCL-mutant were treated with the indicated combinations of a sublytic dose of SLO (500 ng/ml), recombinant GrM (0.5 µM), and/or recombinant GrM-SA (0.5 µM) for 16 h at 37°C. Viable cells were quantified using the methylene blue assay. Data represent the percentages of viable cells as compared to HeLa cells that overexpressed SERPINB4 RCL-mutant and were treated with buffer only, which was set as 100%. Figure represents the mean ± SD of 4 independent experiments; * *p*<0.05. (**D**) HeLa cells stably transfected with SERPINB4 or SERPINB4 RCL-mutant were treated with the indicated combinations of a sublytic dose of SLO (500 ng/ml), recombinant GrM (1 µM), and/or recombinant GrM-SA (1 µM) for 20 h at 37°C. Cell viability was determined using flow cytometry, with AnnexinV and PI negative cells considered viable (mean ± S.D., *n* = 3, ** *p*<0.005). (**E**) HeLa cells stably transfected with SERPINB4 or SERPINB4 RCL-mutant were treated with the indicated combinations of a sublytic dose of SLO (500 ng/ml), recombinant GrM (1 µM), and/or recombinant GrM-SA (1 µM) for 4 h at 37°C. Total cell lysates were immunoblotted using antibodies against nucleophosmin and nm23H-1 (which served as a loading control).

To examine whether SERPINB4 also inhibits the proteolytic activity of GrM in tumor cells, the effect of overexpression of SERPINB4 in HeLa cells on GrM-induced cell death was investigated. First, SERPINB4 RNA and protein expression by HeLa cells stable transfected with SERPINB4, SERPINB4 RCL-mutant, or an empty vector were determined. RT-PCR analysis revealed SERPINB4 RNA expression in both HeLa cells that overexpressed SERPINB4 and SERPINB4 RCL-mutant (Fig. 5A, upper panel). Mock-transfected HeLa cells and HeLa cells transfected with the empty vector did not express SERPINB4 RNA. GAPDH RNA expression served as a control for RNA/cDNA input (Fig. 5A, lower panel). In accordance with the RNA expression, immunoblot analysis revealed SERPINB4 protein expression by both HeLa cells stable transfected with SERPINB4 and SERPINB4 RCL-mutant, and not by mock-transfected HeLa cells or HeLa cells transfected with the empty vector (Fig. 5B). Second, a GrM cell death assay was employed in which the pore-forming protein SLO was used to deliver recombinant GrM into HeLa cells that overexpressed either SERPINB4 or SERPINB4 RCL-mutant. As a control, we included the catalytically inactive GrM mutant (*i.e.* GrM-SA). The methylene blue assay revealed that treatment of the cells with only a sublytic dose of SLO resulted in about 20% reduction of viable cells. As expected, the percentage of viable HeLa cells that overexpressed inactive SERPINB4 RCL-mutant significantly decreased upon treatment with SLO and recombinant GrM, but not with recombinant GrM only, SLO only, or the SLO/GrM-SA combination (Fig. 5C, left panel). In contrast, GrM-induced cell death of HeLa cells was fully rescued by overexpression of SERPINB4 (Fig. 5C, right panel). Similar results were obtained on a single cell level by staining with Annexin V and PI and subsequently FACS analysis. Again, GrM-induced cell death was inhibited by overexpression of SERPINB4 (Fig. 5D).

To further determine whether GrM is inactivated by SERPINB4 after intercellular delivery, cleavage of the preferred GrM-substrate nucleophosmin was investigated. As expected, nucleophosmin was cleaved upon SLO-mediated delivery of GrM, and not GrM-SA, into HeLa cells overexpressing SERPINB4 RCL mutant (Fig. 5D, right lanes). In contrast, GrM-mediated intracellular cleavage of nucleophosmin was inhibited by overexpression of SERPINB4 (Fig. 5D, left lanes), indicating that GrM is inactivated by SERPINB4 upon intracellular delivery.

**Figure 6:**
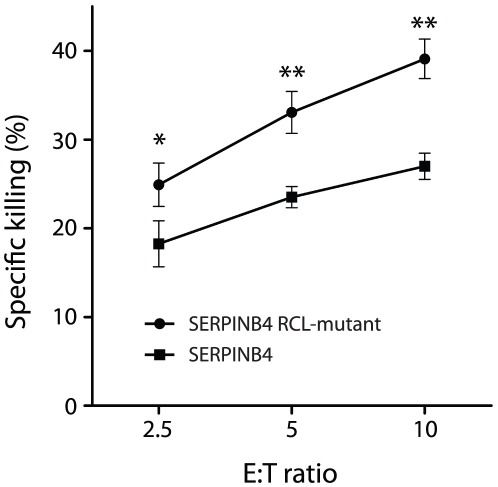
Overexpression of SERPINB4 in HeLa cells inhibits NK cell-mediated cell death. HeLa cells stably transfected with SERPINB4 or SERPINB4 RCL-mutant were loaded with the fluorescent cell staining dye CFDA-SE and co-cultured with KHYG-1 NK cells in varying E∶T ratio's for 16 h at 37°C. Cells were stained with PI and analyzed by flow cytometry. HeLa cells were separated from KHYG-1 cells by gating for CFDA-SE positive cells. Depicted is the percentage of specific cytotoxicity (mean ± SD, *n* = 3, * *p*<0.05, ** *p*<0.005).

In addition to exogenous recombinant GrM-induced cell death, we also investigated whether SERPINB4 inhibits endogenous native GrM-induced cell death. The NK cell line KHYG-1, which is known to express large amounts of GrM and hardly, if any, GrA and GrB [34], was co-cultured in various ratios with HeLa cells stably transfected with either SERPINB4 or SERPINB4 RCL mutant. HeLa cells were killed by KHYG-1 cells in a ratio dependent manner (Fig. 6). In contrast, overexpression of SERPINB4 significantly inhibited KHYG-1 mediated cell death as compared to HeLa cells stably transfected with SERPINB4 RCL mutant. These results indicate that cellular overexpression of SERPINB4 inhibits recombinant GrM-induced as well as NK cell-mediated cell death and that this inhibition is dependent on the RCL of the serpin.

## Discussion

The granule-exocytosis pathway is the major mechanism for cytotoxic lymphocytes to kill tumor and virus-infected cells [1], [2], [3], [4]. Tumor cells can evade GrB-mediated cell death by expression of the intracellular serine protease inhibitor SERPINB9 [17], [18]. However, such physiological intracellular inhibitors are unknown for the other four human granzymes. In mice, GrM is inhibited by the murine serpin SPI-CI, which protects cells from GrM-mediated cell death [35]. SPI-CI is unique for mice and no human orthologue is known. In the present study, we show for the first time that human GrM also has an endogenous intracellular inhibitor that protects cells from GrM-mediated killing. We have demonstrated that GrM cleaves SERPINB4 in its RCL after P1-Leu, resulting in the formation of a typical serpin-protease SDS-stable complex (Figs. 1, 2, 3). Furthermore, SERPINB4 inhibited the proteolytic activity of GrM towards natural macromolecular substrates (Fig. 4 and 5E) and overexpression of SERPINB4 in HeLa cells inhibited recombinant GrM-induced as well as NK-cell mediated cell death (Fig. 5 and 6).

Our findings are consistent with the facts that GrM preferably cleaves after a P1-Leu and, to a lesser extent, after P1-Met [30], [36], and that SERPINB4 harbours a Leu at the P1-position in its RCL [31], [37]. Indeed, mutation of the P2-P1-P1′ triplet in the SERPINB4 RCL completely abolished complex formation with GrM (Fig. 2A) and N-terminal sequencing revealed that GrM cleaved SERPINB4 at least after the Leu residue at the RCL P1-position (Fig. 2C). In addition, protection of HeLa cells against both recombinant GrM-induced and NK cell-mediated cell death by cellular overexpression of SERPINB4 also depended on the RCL of the serpin (Fig. 5 and 6). Although SERPINB4 is the only intracellular serpin that meets the primary specificity of human GrM in that it harbours a Leu or Met amino acid at the putative P1-position in its RCL [32], [38], we cannot fully exclude that other intracellular serpins than SERPINB4 inhibit human GrM. Few serpins are known to use more than one RCL P1-residue to inhibit different target proteases, thereby broadening their inhibitory profile [38]. Interestingly, the GrB-inhibitor SERPINB9 harbours a Met residue at the putative P3′ position in its RCL. Indeed, GrM cleaves SERPINB9, however, an SI value of 59 points to a substrate rather than an inhibitor [30]. Therefore, it has been proposed that cleavage and inactivation of SERPINB9 by GrM clears the way for GrB-induced target cell killing [30]. Whether intracellular serpins other than SERPINB4 exist that inhibit human GrM activity remains an open question.

Whether SERPINB4 is a physiologic inhibitor of GrM remains to be investigated. We showed that SERPINB4 forms a typical serpin-protease SDS-stable complex with both recombinant and native human GrM (Fig. 1). Kinetic analysis of GrM-inhibition by SERPINB4 revealed a SI of 1.6 (Fig. 3A), indicating that about two-third of the SERPINB4 molecules forms a covalent complex with GrM and inhibits its activity, whereas about one-third of the SERPINB4 molecules is a substrate of GrM without trapping the protease. This corresponds with the relative intensity of the bands in Fig. 1A representing complexed and cleaved SERPINB4. The SI for SERPINB4 and GrM is in the similar range as the physiological interaction between SERPINB9 and caspase-1 (SI∼1.7) [39] and somewhat higher as compared to SERPINB9 and GrB (SI∼1.0) [40]. The inhibitory potency of SERPINB4 towards GrM seems different for a small molecular substrate (SI = 1.6; Fig. 3A) as compared to macromolecular substrates (SI>1.6; Fig. 4). The reason for this discrepancy remains unclear, but might be due to our recombinant proteins. It could be that a tiny fraction (<1%) of our recombinant GrM is not properly inhibited by SERPINB4 due to abnormal folding or glycosylation by P. pastoris, but retains its proteolytic activity. If so, this will not influence the determined SI-value significantly (Fig. 3A). However, cleavage of nucleophosmin by GrM is very efficient and occurs already at nanomolar concentrations of GrM [11], therefore a tiny fraction of non-inhibited GrM can be demonstrated by Western blot analysis (Fig. 4). The determined *k_inh_* of 1.3×10^4^ M^−1^s^−1^ for the rate of GrM inhibition by SERPINB4 (Fig. 3B) was into a similar range as compared with other SERPINB4-protease interactions. Previously, SERPINB4 has been demonstrated to inhibit cathepsin G and chymase with *k_inh_*-values of 1.0×10^5^ M^−1^s^−1^ and 2.8×10^4^ M^−1^s^−1^, respectively [41]. The *k_inh_*-value for SERPINB4 and GrM is lower as compared to SERPINB9 and GrB (1.7×10^6^ M^−1^s^−1^) [40]. However, a physiological inhibitory role has been proposed for serpins with a relative low *k_inh_*-value [32], for instance inhibition of TNF-mediated apoptosis by SERPINB4 via cathepsin G (*k_inh_* 1.0×10^5^ M^−1^s^−1^) [26], endogenous anti-inflammatory action of SERPINB9 via inhibition of caspase 1 (*k_inh_* 7×10^2^ M^−1^s^−1^) [39], activated protein C inhibition by Protein C inhibitor (SERPINA5) (*k_inh_* 2.5×10^4^ M^−1^s^−1^) during anti-coagulation [42], and complement C1 and kallikrein inhibition by C1-inhibitor (SERPING1) (*k_inh_* 2.8×10^3^−1.7×10^4^ M^−1^s^−1^) [43]. In this context, it should also be mentioned that, like for other serpins [44], the inhibitory capacity of SERPINB4 towards GrM or other serine proteases could potentially be further enhanced by serpin cofactors, although here we excluded a role for heparin (data not shown). We provide *in vitro* evidence that overexpression of SERPINB4 inhibits both GrM-induced and NK cell-mediated cell death in tumor cells (Fig. 5 and 6). Whether SERPINB4 indeed is a physiological GrM-inhibitor in humans is ultimately determined by local concentrations of GrM and SERPINB4 *in vivo*.

In normal tissue, SERPINB4 is mainly expressed by stratified squamous epithelium of both the upper gastrointestinal tract and the female genitourinary system, and by pseudo-stratified columnar epithelium of the conducting airways [45]. Interestingly, tumors originating from these epithelial tissues also express SERPINB4, *i.e.* squamous cell carcinomas of the cervix [46], head and neck, and lung [45]. Moreover, elevated SERPINB4 levels in patients with squamous cell carcinomas are associated with an advanced stage of tumor progression and poor disease-free survival [47], [48]. This indicates that expression of SERPINB4 is beneficial for tumor cells. Indeed, SERPINB4 inhibits both radiation- and TNF-induced apoptosis in transfected cell lines, probably by inhibition of the p38 MAPK pathway and the proteolytic activity of endogenous cathepsin G, respectively [26], [49]. Our current findings that SERPINB4 binds to human GrM and that cellular overexpression of SERPINB4 inhibits GrM-induced cell death suggest a novel function for SERPINB4, *i.e.* enhancement of tumor progression through interference with the granule-exocytosis cell death pathway of cytotoxic lymphocytes. This intriguing concept has been well studied for SERPINB9-expressing tumor cells that efficiently inhibit GrB-induced cell death [17]. SERPINB9 expression in several types of tumors is associated with a poor clinical outcome of patients [19], [20]. In analogy with SERPINB9, SERPINB4 expression may constitute a novel mechanism by which human squamous cell carcinoma tumor cells evade GrM-mediated cytotoxic lymphocyte-induced cell death.
